# Beyond the Typical Atypical Imaging Features of Leptomeningeal Enhancement: A Case Series

**DOI:** 10.3390/brainsci16070708

**Published:** 2026-06-30

**Authors:** Mohammad Hani, Mohammad Alkhaldi, Hafiz Talha Javed, Vansh Patel, Mohamad Assker, Rucha Bahekar, Parissa Feizi, Ashley E. Catseel, Teryn Lane, Shitiz Sriwastava

**Affiliations:** 1 Department of Neurology, Jackson T. Stephens Spine and Neuroscience Institute, University of Arkansas for Medical Sciences, Little Rock, AR 72205, USA; 2 B.J. Medical College, Ahmedabad 380016, Gujarat, India; 3 Sheikh Khalifa Medical City, Abu Dhabi 51900, United Arab Emirates; 4 Department of Neuroradiology, West Virginia University, Morgantown, WV 26506, USA

**Keywords:** leptomeningeal enhancement, leptomeningeal disease, magnetic resonance imaging, neuroradiology, leptomeningeal carcinomatosis, tuberculous meningitis, neurosarcoidosis, central nervous system lymphoma

## Abstract

**Highlights:**

**What are the main findings?**

- Patterns of leptomeningeal enhancement on MRI brain and spine can be somewhat specific to certain conditions.
- Neurosarcoidosis can mimic a wide variety of other diagnoses.

**What are the implications of the main findings?**

- Understanding nodular, diffuse, smooth, basal cisternal, cranial nerve, and nerve-root leptomeningeal enhancement patterns can help guide the diagnostic workup and narrow the differential diagnosis.
- Neurosarcoidosis should be considered as a differential diagnosis more frequently to avoid misdiagnosis.

**Abstract:**

Background and Purpose: Contrast-enhanced MRI findings of leptomeningeal enhancement (LME) indicate dysfunction of the blood–brain barrier and can be seen in various neoplastic, infectious, and inflammatory conditions. To the best of our knowledge, systematic evaluation of different patterns of LME remains limited. This case series aims to describe and compare MRI patterns of leptomeningeal enhancement across diverse etiologies. Materials and Methods: We retrospectively evaluated six patients with known or suspected leptomeningeal disease on contrast-enhanced MRI studies of the brain and/or spine with regard to pattern, distribution, localization, and thickness of LME. Results: The patterns of neoplastic causes (glioblastoma, lymphoma, and adenocarcinoma) were characterized by diffuse, thick, and nodular LME, as well as frequently affected cranial nerve and nerve-root involvement. The infections caused various patterns: in cases of tuberculous meningitis, LME was focal and basal in nature, accompanied by parenchymal lesions; whereas in cases of HIV-related meningitis, LME was diffuse and exhibited a pseudocisternogram pattern. The inflammation (suspected neurosarcoidosis) revealed smooth-to-nodular LME with spinal and suprasellar involvement. Conclusions: Although LME patterns are not specific, systematic evaluation of enhancement characteristics may provide useful diagnostic clues and assist in narrowing the differential diagnosis.

## 1. Introduction

Leptomeningeal enhancement (LME) represents abnormal contrast enhancement involving the pia-arachnoid complex and subarachnoid space resulting from disruption of the blood–brain barrier and infiltration of the leptomeninges by infectious, inflammatory, or neoplastic processes [1,2]. LME is best visualized on post-contrast T1-weighted and fluid-attenuated inversion recovery (FLAIR) magnetic resonance imaging (MRI) sequences and may involve the cerebral sulci, basal cisterns, cranial nerves, spinal cord, and nerve roots [3,4]. MRI also plays an important role in differentiating leptomeningeal from pachymeningeal enhancement patterns and in identifying associated neuroradiologic features that may provide diagnostic clues regarding the underlying disease process [1,4].

Leptomeningeal enhancement has been described in a broad spectrum of neurologic disorders including leptomeningeal carcinomatosis, lymphoma, tuberculous meningitis, fungal and viral meningitis, and granulomatous inflammatory diseases such as neurosarcoidosis [5,6]. Because presenting clinical manifestations are frequently nonspecific and overlapping, MRI pattern recognition through characterization of enhancement morphology, distribution, localization, thickness, and associated cranial nerve or spinal nerve-root involvement may assist in narrowing differential diagnoses and directing further diagnostic evaluation [5,7,8]. Recognition of characteristic enhancement patterns may facilitate earlier cerebrospinal fluid analysis, targeted tissue sampling, and diagnosis of rapidly progressive central nervous system disease processes associated with substantial morbidity and mortality [6,9].

Distinct MRI enhancement patterns have been associated with specific disease categories. Neoplastic leptomeningeal dissemination commonly demonstrates diffuse linear or nodular enhancement involving the cerebral sulci, cranial nerves, and cauda equina [8,9]. Infectious etiologies such as tuberculous meningitis more frequently demonstrate basal cisternal leptomeningeal enhancement with associated inflammatory exudates, hydrocephalus, and cranial neuropathies [10,11]. Inflammatory and granulomatous disorders including neurosarcoidosis may demonstrate variable leptomeningeal enhancement involving the suprasellar cisterns, optic pathways, spinal leptomeninges, and nerve roots [12,13]. Prior neuroradiologic studies have additionally demonstrated overlap in enhancement morphology and distribution among infectious, inflammatory, and neoplastic etiologies, particularly in patients presenting with cranial neuropathies, encephalopathy, hydrocephalus, multifocal neurologic deficits, or myeloradiculopathy [1,5].

The current literature predominantly focuses on isolated disease-specific imaging findings rather than comparative characterization of MRI leptomeningeal enhancement patterns across diverse etiologies. By correlating enhancement morphology and distribution with underlying disease processes, this case series aims to highlight clinically relevant MRI features that may improve interpretation of complex leptomeningeal disease presentations and assist in narrowing differential diagnoses in challenging neurologic cases. This retrospective case series presents six patients with distinct neoplastic, infectious, and inflammatory causes of leptomeningeal enhancement and emphasizes the diagnostic value of systematic MRI pattern evaluation.

## 2. Materials and Methods

This retrospective single-center case series included six patients with leptomeningeal enhancement (LME) evaluated at the University of Arkansas for Medical Sciences (UAMS). Clinical records and contrast-enhanced magnetic resonance imaging (MRI) studies of the brain and/or spine were retrospectively reviewed. The clinical dataset included neurologic presentation, cerebrospinal fluid (CSF) findings, microbiologic and pathologic data when available, treatment course, and clinical outcomes. MRI studies were reviewed by board-certified neurologists with expertise in neuroimaging, and imaging findings were assessed by consensus based on enhancement morphology, distribution, thickness, cranial nerve involvement, and associated parenchymal abnormalities. Imaging findings were correlated with the underlying clinical diagnosis and previously described neuroradiologic patterns reported in the literature.

The cases represented diverse neoplastic, infectious, and inflammatory/granulomatous etiologies of leptomeningeal disease, including glioblastoma with leptomeningeal dissemination, lymphoma, metastatic gastrointestinal adenocarcinoma, tuberculous meningitis, HIV-associated meningitis, and possible neurosarcoidosis. Particular attention was given to MRI enhancement characteristics and their diagnostic relevance across varying disease processes. This case series was approved by the University of Arkansas for Medical Sciences Institutional Review Board (IRB #300007). Informed consent was obtained when applicable, and all patient information was de-identified to maintain confidentiality in accordance with institutional ethical standards.

## 3. Case Series

### 3.1. Case 1: Spindle Cell Neoplasm/Glioblastoma

A 76-year-old man with a history of hypertension initially developed progressive bilateral lower extremity weakness and recurrent falls beginning in November 2021, for which he was diagnosed with subacute polyneuropathy. Despite treatment with two courses of intravenous immunoglobulin and corticosteroids, his symptoms continued to worsen, with progression to paraplegia and urinary retention, prompting readmission for further evaluation. Neurologic examination revealed severe bilateral lower extremity weakness (0/5 proximally), areflexia in the lower extremities, a sensory level to approximately T10, and bilateral lower motor neuron facial nerve (CN VII) palsies (right greater than left), along with mild encephalopathy and upper extremity incoordination. MRI of the neuroaxis demonstrated diffuse nodular thickening and avid leptomeningeal enhancement of the cauda equina nerve roots, diffuse dural enhancement along the cervicothoracic spinal cord, and focal nodular enhancement in the distal thoracic spine (Figure 1). Additional findings included intramedullary T2 hyperintensity in the cervical and thoracic cord with subtle enhancement, as well as enhancement of multiple cranial nerves (including CN V, VII, and VIII) and a right temporal dural-based lesion. CSF analysis revealed lymphocytic pleocytosis (WBC 38), markedly elevated protein (281 mg/dL), and low glucose, but repeated infectious, autoimmune, and paraneoplastic evaluations including ANA, ANCA, aquaporin-4, demyelinating panels, infectious studies, and cytology were unrevealing.

Given the progressive myeloradiculopathy with multifocal cranial neuropathies and diffuse leptomeningeal and dural enhancement, a neoplastic process was favored over inflammatory or granulomatous etiologies after failure of immunomodulatory therapies, including IVIG, steroids, and plasma exchange. PET-CT demonstrated mild metabolic activity corresponding to areas of spinal canal enhancement but did not identify a clear systemic primary malignancy. Due to continued clinical decline and nondiagnostic CSF studies, the patient underwent thoracolumbar dural biopsy. Histopathology revealed a spindle cell neoplasm, and subsequent molecular profiling supported a diagnosis of glioblastoma, IDH-wildtype (WHO grade 4), mesenchymal subtype. This case illustrates an atypical presentation of diffuse leptomeningeal and dural-based enhancement with nodular thickening involving the cauda equina, spinal cord, and cranial nerves, ultimately representing disseminated high-grade primary CNS malignancy.

### 3.2. Case 2: Lymphoma

A 61-year-old man with relapsed T-cell/histiocyte-rich large B-cell lymphoma, previously treated with BEAM autologous stem cell transplantation (2/2020) and CAR-T therapy (14 January 2025, with subsequent boost on 24 February 2025), presented with progressive bilateral lower extremity weakness, impaired ambulation, and weight loss while undergoing treatment with glofitamab. His prior neurologic history included seizures with EEG demonstrating moderate encephalopathy and possible right temporal subclinical seizure activity. On current presentation, examination revealed impaired cognition (oriented to person and place), symmetric lower extremity weakness (approximately 3/5), distal sensory loss to light touch up to the knees, and areflexia. He also exhibited marked left-sided proptosis with ophthalmoplegia, which was attributed to thyroid eye disease with partial cranial nerve III and VI palsies, with normal MRI brain and orbits and negative acetylcholine receptor antibodies.

Given concern for central nervous system involvement, lumbar puncture demonstrated pleocytosis (WBC 35/mm^3^) with cytology and flow cytometry identifying a small kappa light chain–restricted CD19/CD20-positive B-cell population and rare atypical lymphoid cells, consistent with leptomeningeal involvement by lymphoma. Serial CSF studies demonstrated persistent atypical cells with improving pleocytosis (WBC 17 → 4 following treatment), and subsequent cytology was negative for malignant cells. CSF analysis showed markedly elevated protein with a dynamic trend (2300 → 3100 → 312 → 17 mg/dL) and variable glucose levels (91 → 138 → 36 → 53 mg/dL) as the treatment progressed. Broad autoimmune and inflammatory workup, including autoimmune encephalitis panel, ANA, ANCA, ACE, and serum lysozyme, was unrevealing. MRI of the spine revealed extensive thick leptomeningeal enhancement involving the distal conus, lumbar nerve roots, and sacral regions, consistent with neoplastic meningeosis (Figure 2). The patient was treated with systemic steroids, methotrexate and rituximab along with twice-weekly intrathecal chemotherapy (cytarabine and methotrexate) via an Ommaya reservoir, with a course complicated by significant pancytopenia requiring frequent transfusions.

Despite aggressive management, the patient experienced continued clinical decline with persistent neurologic deficits. Given his refractory lymphoma and inability to tolerate further chemotherapy, goals of care were transitioned to comfort-focused measures, and he was enrolled in inpatient hospice. He passed away two days later.

### 3.3. Case 3: Gastric Adenocarcinoma with Metastasis

A 42-year-old man with a history of hypertension, diabetes mellitus, prior tobacco and methamphetamine use, and recently diagnosed metastatic esophageal adenocarcinoma initially presented with progressive dysphagia, decreased appetite, and approximately 40-pound weight loss over six months. Diagnostic workup revealed extensive lymphadenopathy on CT imaging, and subsequent EGD/EUS identified a distal esophageal mass with biopsy confirming adenocarcinoma and metastatic involvement of perigastric lymph nodes. He was discharged with outpatient follow-up but was readmitted shortly thereafter with progressive neurologic symptoms, including slurred speech, generalized weakness, ataxia, and intractable nausea and vomiting. Neurologic examination was notable for scanning dysarthria, left facial nerve (CN VII) palsy, right-beating horizontal and downbeat nystagmus, truncal and gait ataxia, and bilateral upper extremity dysmetria, localizing to posterior fossa and brainstem involvement. Initial CT head was unremarkable; however, MRI brain with contrast demonstrated diffuse, thick leptomeningeal enhancement involving the bilateral cerebellar folia, cerebellar vermis, brainstem surface, Sylvian fissures, suprasellar and basal cisterns, and anterior temporal sulci, with additional involvement of multiple cranial nerves (V, VII, VIII) and skull base foramina (Figure 3). Associated vasogenic edema in the cerebellar vermis led to early obstructive hydrocephalus with dilation of the lateral and third ventricles. Due to the presence of hydrocephalus and risk of herniation, lumbar puncture was deferred.

The patient was admitted to the neurocritical care unit for close monitoring of worsening hydrocephalus, where an external ventricular drain (EVD) was placed, followed by ventriculoperitoneal (VP) shunt placement. Despite these interventions, his hospital course was marked by rapid neurologic and systemic decline. He developed acute hypoxic respiratory failure following a suspected aspiration event requiring intubation and subsequently demonstrated electrographic seizures with temporal onset on EEG, managed with escalation of antiseizure therapy including lacosamide, lorazepam, and topiramate. His course was further complicated by acute ischemic stroke and gastrointestinal bleeding requiring transfusions. Given the extent of diffuse leptomeningeal metastasis, poor neurologic prognosis, and a lack of meaningful therapeutic options, goals-of-care discussions were held with the family. The decision was made to transition to comfort-focused care, and the patient was compassionately extubated, passing away later that day.

### 3.4. Case 4: Tuberculosis

A 33-year-old South Asian male with no known past medical history presented to the ED with a 2-week history of fever, sore throat, non-productive cough, and runny nose. He was seen at a different facility, given symptomatic treatment and amoxicillin–clavulanate. Three days prior to his visit, he became confused and developed mood disturbances and abnormal behavior, which was noted by his friends, who took him to the hospital where he escaped and went missing for a day. He was later found confused and had multiple episodes of vomiting associated with a severe headache. No witnessed seizure activity or aggressive behavior was reported. His last travel was 4 years ago to his home country, but nothing more recent. He lived with seven roommates. On examination he was confused, disoriented, and unaware of his surroundings; however, he was vitally stable and afebrile. He had positive nuchal stiffness, Brudzinski’s and Kernig’s sign; but no rashes of note. He was started on empiric ceftriaxone, vancomycin, and acyclovir. CT head was unremarkable. LP was clear, with a nucleated cell count of 43 × 10^6^/L, protein of 1.39 g/L, and glucose of 0.8 mmol/L. A sputum acid fast bacilli smear was negative. CXR showed faint nodular infiltrates in bilateral upper and mid lung zones. He was admitted as a case of bacterial meningitis and pneumonia. Shortly after admission, the patient became hypotensive and bradycardic, and his GCS dropped to 11 and was therefore transferred to the ICU. Repeat CT head with contrast revealed left frontal basal and anteroinferior insular/ganglionic hypodensities; however, there were no leptomeningeal or pachymeningeal changes or enhancement of note. CSF culture later revealed rifampicin-resistant TB, and the patient was started on moxifloxacin, linezolid, cyclosporine, and clofazimine. An MRI brain with contrast was done following a series of head CTs, and it confirmed the left basal ganglionic and insular lesions (Figure 4). Additionally, MRI revealed scattered brain parenchymal enhancing foci as well as localized leptomeningeal enhancement in the left insular fissure. He received a total of 8 weeks of daily dexamethasone, improved and was discharged home to continue his anti-TB regimen for a year.

### 3.5. Case 5: HIV Meningitis

A 70-year-old man with a history of diabetes mellitus, hypertension, dyslipidemia, end-stage renal disease on hemodialysis, peripheral vascular disease, ischemic heart disease post-percutaneous cardiac intervention, and HIV since 1999 on treatment with a CD4 count of 209 and a CD4/CD8 ratio of 0.61 presented to the ED with subjective chills and fever for the past few days, productive cough with blood-tinged white sputum, and five episodes of hemoptysis during his ED visit, with a notable BP of 200/100. He was admitted as a case of hypertensive urgency with pulmonary hemorrhage versus atypical pneumonia. He underwent bronchoscopy with bronchoalveolar lavage, which was negative for TB and PCP; sputum cultures grew klebsiella pneumonia which was treated appropriately. Several days later, the patient developed an altered mental status where his GCS dropped to 9 and developed stridor and signs of bulbar palsy (frequent choking and difficulty swallowing). The CT head was unremarkable. LP was cloudy, with a nucleated cell count of 425^6^/L, protein of 1.36 g/L, and glucose of 3.6 mmol/L (blood glucose on the same day was 16.5 mmol/L) with no positive cultures. He was transferred to the ICU for intubation; his antibiotic course was upgraded to meropenem, vancomycin, ceftriaxone, and acyclovir to cover for meningitis. An MRI of the brain performed the next day revealed occipital horn diffuse signal changes compatible with ventriculitis and stable hydrocephalus (Figure 5). EEG demonstrated severe diffuse background slowing suggestive of severe diffuse encephalopathy but no epileptiform discharges or electrographic seizures.

Ten days later the patient’s GCS dropped to 4. The patient underwent a tracheostomy, and a repeat MRI was performed, which revealed confluent diffuse posterior fossa, bilateral insular fissural, and basal CSF leptomeningeal enhancement with a pseudocisternogram appearance suggestive of severe meningeal inflammation and contrast leakage. Development of a new pontine hemorrhagic infarct was noted; clopidogrel and heparin prophylaxis were held and switched to mechanical thromboprophylaxis.

One day later dilated fixed pupils bilaterally were noted, and CT head was done revealing new left parietal and left thalamic hemorrhagic stroke with intraventricular and subarachnoid extension, as well as worsening of the brainstem hemorrhagic infarct and diffuse brain edema with progressing hydrocephalus. Neurosurgical intervention was deferred due to futility. The next day, the patient developed sudden cardiac arrest (pulseless electrical activity) and CPR was performed as per ACLS. The patient was pronounced dead after 40 min of cardiopulmonary resuscitation.

### 3.6. Case 6: Possible Neurosarcoidosis

A 29-year-old man with no significant past medical history presented to the ED reporting a sudden onset of sharp lower back, bilateral hip, and knee pain 2 months prior to admission by non-ascending numbness and weakness in bilateral lower extremities. Strength continued to worsen and a week prior to admission, he developed difficulty with micturition and defecation. Neurological examination was notable for symmetric proximal more than distal lower extremity weakness, loss of sensation to light touch and pinprick bilaterally up to the knees, absent patellar and Achilles reflexes, and upgoing plantar responses.

Investigations included contrasted MRIs of the neuroaxis which revealed diffuse smooth-to-nodular leptomeningeal enhancement involving the lower thoracic cord, conus medullaris, cauda equina, and suprasellar cistern along the optic complex and the infundibulum (Figure 6). A lumbar puncture demonstrated clear, xanthochromia cerebrospinal fluid (CSF) with a marked lymphocytic pleocytosis (231 WBC/mm^3^), extremely elevated protein (742 mg/dL), and hypoglycorrhachia (glucose 15 mg/dL). Initial CSF infectious studies, including Gram stain, bacterial cultures, and meningitis/encephalitis PCR panel, were negative. Given the concern for fungal meningitis versus other inflammatory or neoplastic processes, the patient was empirically started on amphotericin B, high-dose methylprednisolone, and intravenous immunoglobulin (IVIG) by the Infectious Disease and Neurology teams. He showed mild neurological improvement during his hospitalization but was discharged with persistent lower extremity weakness and incontinence, requiring a rolling walker and ongoing outpatient workup for definitive etiology. Fungal workup came back unremarkable. GAD65 Antibody assay was 0.18 nmol/L on the serum autoimmune/paraneoplastic encephalopathy panel but was deemed incidental (Table 1).

## 4. Discussion

The first case illustrates an atypical presentation of diffuse leptomeningeal and dural-based enhancement with nodular thickening involving the cauda equina, spinal cord, and cranial nerves, ultimately representing disseminated high-grade primary CNS malignancy without a primary parenchymal tumor. Studies have shown different incidences of leptomeningeal spread of primary CNS malignancies. The most recent studies found in the medical literature show that the incidence of LMS was 11.4% at initial diagnosis (Park et al. 2024) and 10.8% at recurrence (Park et al. 2025) [14,15]. Leptomeningeal metastasis seen in glioblastoma does not have any specific appearance. It can be focal or diffuse, linear or nodular and can be present intra- and extracranially [16]. It is reported to spread almost equally throughout the cervical, thoracic, and lumbar spine levels [17]. Up to 38% of cases involve the cauda equina or conus medullaris. Intracranial metastasis has also been described [16].

Leptomeningeal involvement in systemic lymphoma is well documented in the literature but reported as a rare late complication with an incidence varying widely depending on the histologic subtype, from extremely rare to more than 25% in highly aggressive subtypes. However, data describing specific MRI patterns of LME in CNS lymphoma remain limited. The study described leptomeningeal thickening and enhancement in cranial nerves, the spinal cord and nerve roots [18]. In our case MRI of the spine revealed extensive thick leptomeningeal enhancement involving the distal conus, lumbar nerve roots, and sacral regions.

While leptomeningeal disease secondary to gastroesophageal carcinoma is rare, it is well documented in the literature and is considered a very poor prognostic factor. As described in Lukas et al.’s case series and systematic review of seven cases of leptomeningeal carcinomatosis due to esophageal cancer and Baccili Cury Megid et al.’s retrospective cohort study of 3283 patients with gastroesophageal carcinoma, the adenocarcinoma subtype was the most common to metastasize to the leptomeninges [19,20]. While these articles focused on clinical outcomes, unfortunately, they did not describe any imaging patterns specific to esophageal adenocarcinoma. A case report by Lu et al. described detection of leptomeningeal metastasis from gastroesophageal junction adenocarcinoma on an 18F-FDG PET/CT showing abnormal hypermetabolism in the cerebellum [21]. Additionally, posterior fossa and leptomeningeal involvement have been described in metastatic gastrointestinal and gynecologic malignancies [22]. In our case there was diffuse, thick leptomeningeal enhancement involving the bilateral cerebellar folia, cerebellar vermis, brainstem surface, Sylvian fissures, suprasellar and basal cisterns, and anterior temporal sulci, with additional involvement of multiple cranial nerves (V, VII, VIII) and skull base foramina.

The pathogenesis of CNS tuberculosis is thought to follow a two-step model in which the bacteria from the initial pulmonary infection spread to oxygen-rich areas in the CNS including the meninges, subpial or subependymal regions of the brain, or the spinal cord forming caseating foci also known as Rich foci. They can then rupture into the ventricles or the subarachnoid space leading to meningitis [23]. On MR imaging, the most common and relatively specific finding is enhancing exudate in the basal cisterns [11]. The meningeal enhancement pattern is described as thick, irregular and occasionally nodular. Other imaging findings include hydrocephalus, cranial nerve involvement, and vasculitis, all of which are due to exudate-related inflammation. Patients can also have granulomas (tuberculomas) on imaging which can be caseating or noncaseating. Noncaseating granulomas show T1 and T2 hypointensity and uniform post-contrast enhancement while caseating granulomas typically have T2 hyperintensity and ring-like enhancement due to central liquefication [18].

Direct HIV meningitis does not commonly cause leptomeningeal enhancement. One study of 13 patients with HIV meningoencephalitis found LME in one of eight patients who underwent MR imaging [24]. However, 21% of patients with HIV were found to have LME in another study, which was likely because of other infections due to immunocompromise [25]. Viral meningitis in general can rarely cause LME which is described as subtle, thin, linear, and smooth, which can become more evident if there is concurrent encephalitis. Contrast-enhanced FLAIR was found to be more sensitive than T1-contrasted MR imaging. In contrast, about 50% of pyogenic meningitis cases can have LME on imaging which looks similar but slightly thicker and may demonstrate diffusion restriction on DWI sequences [18].

Approximately 40% of patients with neurosarcoidosis demonstrate LME on MRI. It is the most common finding and frequently affects the skull base and may extend to the spinal cord meninges which can resemble the distribution of lymphoma or tuberculosis. It may appear diffuse or nodular. Parenchymal lesions, dural involvement, vasculitis, and cranial nerve involvement are common findings [18]. In a retrospective study of one hundred patients with neurosarcoidosis to look at imaging patterns, non-enhancing white matter lesions were the most common finding (56%), followed by leptomeningeal enhancement (47%) and pachymeningeal enhancement (32%) [26]. Although our patient demonstrated imaging findings resembling reported neurosarcoidosis patterns, a definitive diagnosis was not established, and the case was therefore classified as possible neurosarcoidosis.

Despite representing markedly different underlying etiologies, several cases in our series demonstrated overlapping MRI appearances, particularly involving diffuse basal cisternal enhancement, cranial nerve involvement, and multifocal spinal leptomeningeal disease. Similar enhancement patterns were observed across neoplastic, infectious, and inflammatory conditions, often making distinction based on enhancement morphology alone difficult [5,18]. Notably, thick nodular enhancement with extensive neuraxis involvement was not exclusive to neoplastic disease in our series, while smooth or diffuse leptomeningeal enhancement also occurred in inflammatory and infectious etiologies involving the skull base and spinal leptomeninges [11,12,18]. These findings highlight that associated imaging features, anatomic distribution, and clinicoradiologic correlation may provide greater diagnostic value than the enhancement pattern alone, particularly in cases with overlapping cranial nerve, cauda equina, or posterior fossa involvement [5,6].

## 5. Limitations

Limitations include the retrospective single-center design and small number of cases with heterogeneous underlying etiologies. Several imaging patterns demonstrated substantial overlap across neoplastic, infectious, and inflammatory processes, limiting the specificity of MRI appearance alone in establishing a definitive diagnosis. Histopathologic confirmation was not available in every case, and MRI protocols were not completely uniform across all patients. Additionally, the descriptive nature of the series limited quantitative comparison of enhancement characteristics between disease entities.

## 6. Conclusions

This case series highlights the broad and frequently overlapping MRI appearances of leptomeningeal enhancement across neoplastic, infectious, and inflammatory disease processes. Despite markedly different underlying etiologies, several cases demonstrated similar enhancement morphology and neuraxis distribution, creating important diagnostic challenges and potential imaging pitfalls. Our findings emphasize that interpretation of leptomeningeal disease should extend beyond the enhancement pattern alone and incorporate associated imaging findings, anatomic distribution, and clinicoradiologic correlation. Recognition of these overlapping yet clinically meaningful imaging patterns may help improve diagnostic narrowing, guide earlier targeted workup or biopsy consideration, and support more timely clinical management in complex cases of leptomeningeal disease. By presenting a broad spectrum of neoplastic, infectious, and inflammatory etiologies within a single case series, this report provides a practical educational framework that may assist clinicians and radiologists in recognizing important imaging clues and formulating more informed differential diagnoses in patients presenting with leptomeningeal enhancement.

## Figures and Tables

**Figure 1:**
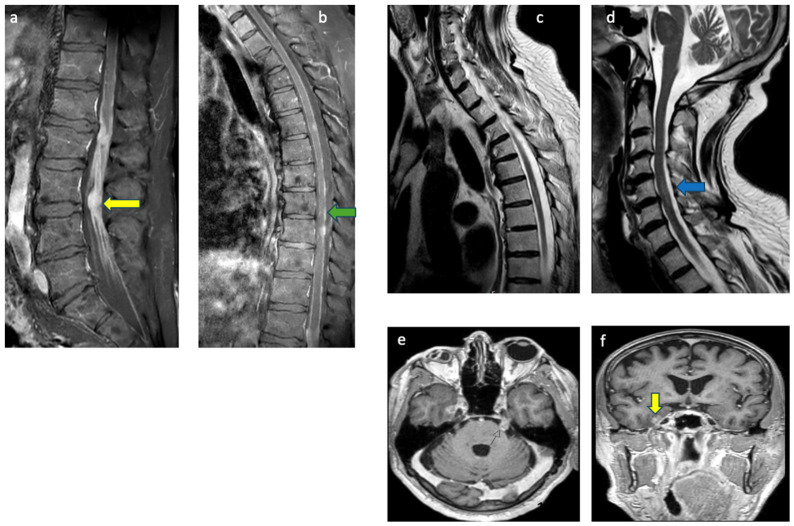
(**a**) Sagittal T1+C MRI of the lumbar spine demonstrates diffuse nodular thickening and avid leptomeningeal enhancement of the cauda equina nerve roots (yellow arrow). (**b**) Sagittal T1+C MRI of the thoracic spine shows diffuse dural enhancement along the cervicothoracic spinal cord, and focal nodular enhancement in the distal thoracic spine (green arrow). (**c**,**d**) Sagittal T2 MRI of the thoracic and cervical spine showing intramedullary T2 hyperintensity in the thoracic and cervical cord respectively (blue arrow). (**e**) Axial T1+C MRI of the brain showing enhancement of the 5th cranial nerve. (**f**) Coronal T1+C MRI of the brain showing a right temporal dural-based lesion with contrast enhancement (yellow arrow).

**Figure 2:**
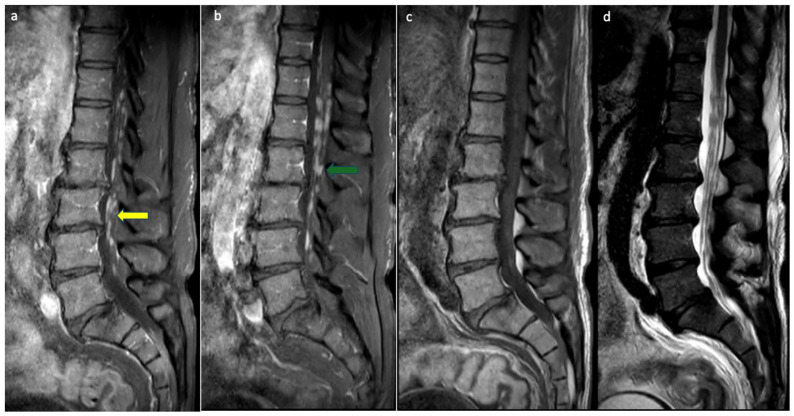
(**a**,**b**) Different cuts of T1+C sagittal MRI lumbar spine showing extensive areas of abnormal enhancement in the nerve roots. These are thickened irregular and show clumping (yellow and green arrow). Additional abnormal enhancement is noted in the thecal sac at the S2 and S3 vertebral body levels. (**c**) T1 sagittal MRI lumbar spine without contrast showing the same cut as (**a**). (**d**) T2 sagittal MRI lumbar spine showing the same cut as (**b**).

**Figure 3:**
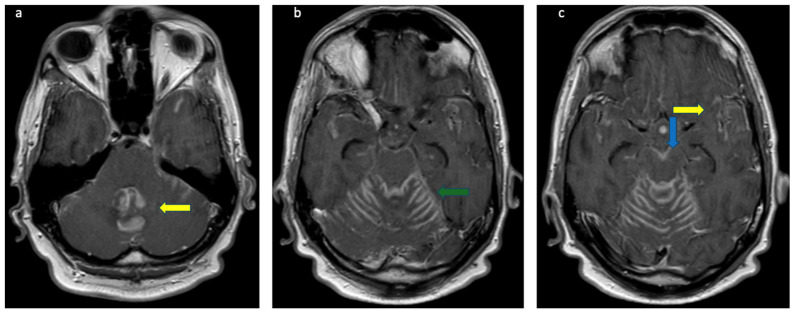
Panels (**a**–**c**) show different cuts of axial T1+C MRI brain demonstrating diffuse, thick leptomeningeal enhancement involving the bilateral cerebellar vermis, cerebellar folia, brainstem surface (yellow, green and blue arrow respectively), Sylvian fissures, suprasellar and basal cisterns, and anterior temporal sulci (blue arrow).

**Figure 4:**
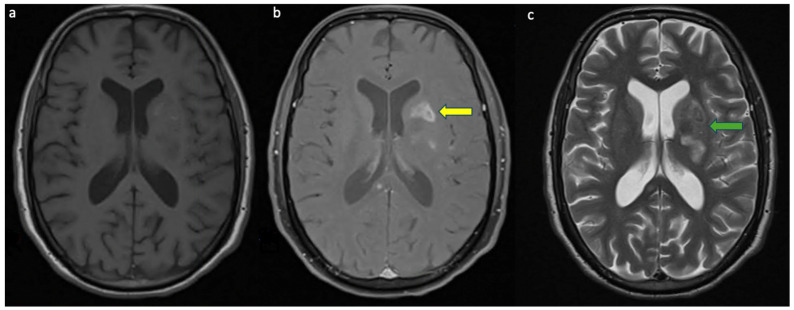
Axial MRI brain revealed scattered brain parenchymal enhancing foci as well as localized leptomeningeal enhancement in the left insular fissure. (**a**) T1 without contrast, (**b**) T1+C (yellow arrow), (**c**) T2 (green arrow).

**Figure 5:**
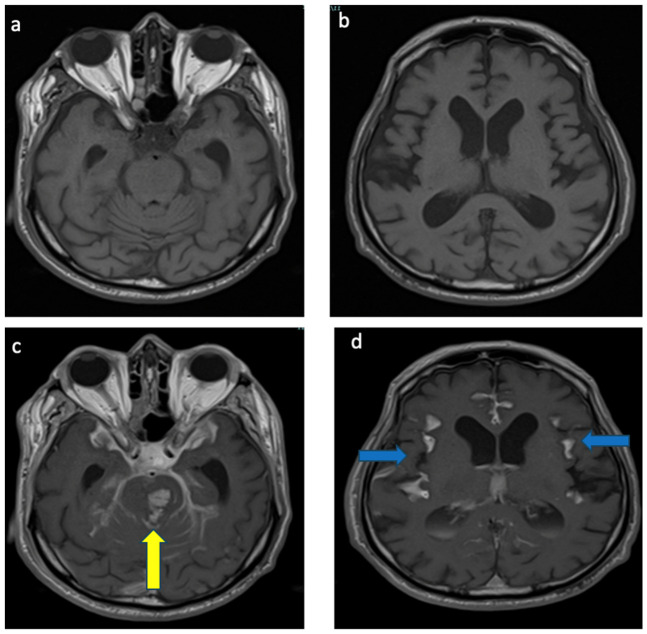
(**a**,**b**) Axial MRI brain T1 images without contrast. (**c**,**d**) Axial MRI brain T1 images with contrast showing confluent diffuse posterior fossa (yellow arrow), bilateral insular fissure (blue arrow), and basal CSF leptomeningeal enhancement (yellow arrow) with pseudocisternogram appearance.

**Figure 6:**
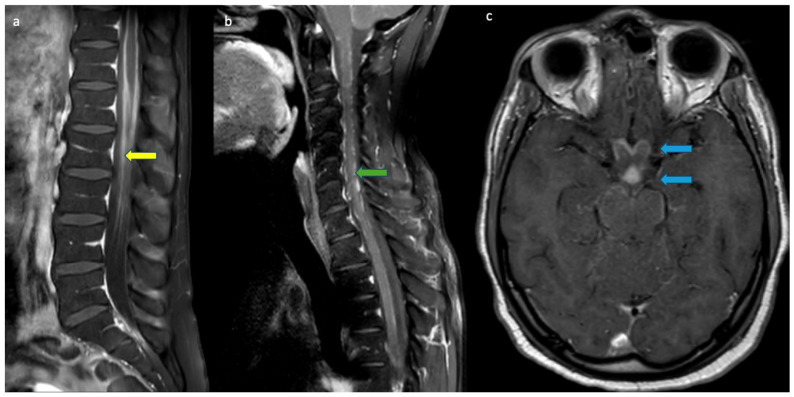
(**a**) Sagittal MRI lumbar spine T1+C: diffuse, smooth-to-nodular leptomeningeal enhancement involving the lower thoracic cord, conus medullaris, cauda equina (yellow arrow). (**b**) Sagittal MRI cervical spine T1+C: diffuse smooth-to-nodular leptomeningeal enhancement involving the cervical and thoracic spinal cord (green arrow). (**c**) Axial MRI brain T1+C: leptomeningeal enhancement is noted, most prominent in the suprasellar cistern along the optic complex and the infundibulum (blue arrow).

**Table 1:** Summary of MRI leptomeningeal enhancement patterns and associated etiologies.

Case	LME Pattern	Similar or Different to the Literature
Spindle cell neoplasm with molecular features consistent with glioblastoma, IDH-wildtype	• Diffuse nodular leptomeningeal enhancement; • Cauda equina nerve-root thickening; • Cervicothoracic dural enhancement; • Cranial nerve enhancement (CN V, VII, VIII); • Intramedullary spinal cord signal abnormality.	Similar pattern to what is reported in the literature for primary CNS neoplasm leptomeningeal metastasis.
2. DLBCL	• Thick leptomeningeal enhancement; • Distal conus involvement; • Lumbar nerve-root enhancement; • Sacral leptomeningeal involvement.	Reported leptomeningeal enhancement and thickening but no specific patterns are described in the literature.
3. GI adenocarcinoma with LM carcinomatosis	• Diffuse thick leptomeningeal enhancement; • Cerebellar folia and vermis involvement; • Brainstem surface enhancement; • Basal cistern and suprasellar involvement; • Multiple cranial nerve involvement.	Similar to previously reported descriptions of leptomeningeal carcinomatosis in the literature. Although there are relatively few reported cases of gastrointestinal adenocarcinoma with leptomeningeal carcinomatosis, the imaging appearance was typical.
4. TB	• Localized leptomeningeal enhancement; • Left insular fissure involvement; • Associated parenchymal enhancing lesions.	Similar to what is seen in the literature.
5. HIV	• Diffuse posterior fossa enhancement; • Bilateral insular fissure enhancement; • Basal CSF leptomeningeal enhancement; • Pseudocisternogram appearance.	More extensive than typically reported in HIV meningitis and more closely resembling patterns observed in severe pyogenic meningitis.
6. Possible neurosarcoidosis	• Smooth-to-nodular leptomeningeal enhancement; • Lower thoracic cord involvement; • Conus medullaris and cauda equina involvement; • Suprasellar cistern involvement; • Optic pathway/infundibular enhancement.	Similar to what is described in the literature.

## Data Availability

The data presented in this case series are available from the corresponding author upon reasonable request. The data are not publicly available because they contain de-identified patient information and are subject to institutional ethical and privacy restrictions.

## Abbreviations

LME	leptomeningeal enhancement
CNS	central nervous system
CSF	cerebrospinal fluid
MRI	magnetic resonance imaging
FLAIR	fluid-attenuated inversion recovery
CN	cranial nerve
IVIG	intravenous immunoglobulin
EEG	electroencephalography
PET-CT	positron emission tomography–computed tomography
EVD	external ventricular drain
VP	ventriculoperitoneal
TB	tuberculosis
DWI	diffusion-weighted imaging
UAMS	University of Arkansas for Medical Sciences

## References

[1] Yogendra P.M., Nickalls O.J., Ho C.L. (2025). Leptomeningeal masses or masquerades: A spectrum of diseases with leptomeningeal enhancement and their mimics. Curr. Med. Imaging.

[2] Marchi L., Mariottini A., Viti V., Bianchi A., Nozzoli C., Repice A.M., Boncompagni R., Ginestroni A., Damato V., Barilaro A. (2024). Leptomeningeal enhancement in multiple sclerosis: A focus on patients treated with hematopoietic stem cell transplantation. Front. Neurol..

[3] Galassi W., Phuttharak W., Hesselink J.R., Healy J.F., Dietrich R.B., Imbesi S.G. (2005). Intracranial meningeal disease: Comparison of contrast-enhanced MR imaging with fluid-attenuated inversion recovery and fat-suppressed T1-weighted sequences. Am. J. Neuroradiol..

[4] Smirniotopoulos J.G., Murphy F.M., Rushing E.J., Rees J.H., Schroeder J.W. (2007). Patterns of contrast enhancement in the brain and meninges. Radiographics.

[5] Sener U., Wilcox J.A., Boire A.A. (2025). Leptomeningeal disease: Current approaches and future directions. Curr. Neurol. Neurosci. Rep..

[6] Sener U., Kumthekar P., Boire A. (2021). Advances in the diagnosis, evaluation, and management of leptomeningeal disease. Neurooncol. Adv..

[7] Saremi F., Helmy M., Farzin S., Zee C.S., Go J.L. (2005). MRI of cranial nerve enhancement. Am. J. Roentgenol..

[8] Hinke M., Skovran A., Dusini N., Azim S. (2022). Leptomeningeal carcinomatosis: A case report and literature review. Cureus.

[9] Barbour A.B., Kotecha R., Lazarev S., Palmer J.D., Robinson T., Yerramilli D., Yang J.T. (2024). Radiation therapy in the management of leptomeningeal disease from solid tumors. Adv. Radiat. Oncol..

[10] Khatri G.D., Krishnan V., Antil N., Saigal G. (2018). Magnetic resonance imaging spectrum of intracranial tubercular lesions: One disease, many faces. Pol. J. Radiol..

[11] Sanei Taheri M., Karimi M.A., Haghighatkhah H., Pourghorban R., Samadian M., Delavar Kasmaei H. (2015). Central nervous system tuberculosis: An imaging-focused review of a reemerging disease. Radiol. Res. Pract..

[12] Jiao L., Wei M., Fu Y. (2022). Characteristics of patients with meningeal carcinomatosis combined with myelopathy. Neuropsychiatr. Dis. Treat..

[13] Msherghi A., Glue-Monroe M., WEldaya R., Pirhoushiaran M., Al Qudah H., Taslicay C.A., Alizada S., Salim H.A., Ballester L.Y., Wintermark M. (2026). Preliminary assessment of an MRI-based grading system for leptomeningeal disease: An exploratory prognostic framework. Neuroradiology.

[14] Park Y.W., Jang G., Kim S.B., Choi K., Han K., Shin N.Y., Ahn S.S., Chang J.H., Kim S.H., Lee S.K. (2024). Leptomeningeal metastases in isocitrate dehydrogenase-wildtype glioblastomas revisited: Comprehensive analysis of incidence, risk factors, and prognosis based on post-contrast fluid-attenuated inversion recovery. Neuro Oncol..

[15] Park Y.W., Jang G., Kim S.B., Han K., Shin N.Y., Ahn S.S., Chang J.H., Kim S.H., Jain R., Lee S.K. (2025). Leptomeningeal metastases at recurrence in IDH-wildtype glioblastomas: Incidence, risk factors, and prognosis based on postcontrast FLAIR imaging. Eur. Radiol..

[16] Birzu C., Tran S., Bielle F., Touat M., Mokhtari K., Younan N., Psimaras D., Hoang-Xuan K., Sanson M., Delattre J.Y. (2020). Leptomeningeal spread in glioblastoma: Diagnostic and therapeutic challenges. Oncologist.

[17] Ginat D.T., Schaefer P.W. (2014). Imaging guidelines and findings of extracranial glioblastoma. J. Neurooncol..

[18] Akcicek E., Aldur A.E., Gocmen R. (2025). Differentiating leptomeningeal diseases through enhancement patterns: A pictorial essay. Neuroradiol. J..

[19] Lukas R.V., Mata-Machado N.A., Nicholas M.K., Salgia R., Antic T., Villaflor V.M. (2015). Leptomeningeal carcinomatosis in esophageal cancer: A case series and systematic review of the literature. Dis. Esophagus.

[20] Megid T.B.C., Baskurt Z., Ma L.X., Barron C.C., Farooq A., Saltiel M.P., Wang X., Bach Y., Ayoama H., Jang R.W. (2024). Leptomeningeal carcinomatosis and brain metastases in gastroesophageal carcinoma: A real-world analysis of clinical and pathologic characteristics and outcomes. J. Neurooncol..

[21] Lu Y., Wang L., Ajani J.A. (2020). Rare esophageal leptomeningeal metastases detected on 18F-FDG PET/CT. Clin. Nucl. Med..

[22] Ivanidze J., Shih R.Y., Utukuri P.S., Ajam A.A., Auron M., Chang S.M., Jordan J.T., Kalnins A., Kuo P.H., Expert Panel on Neurological Imaging (2025). ACR Appropriateness Criteria® brain tumors. J. Am. Coll. Radiol..

[23] Patkar D., Narang J., Yanamandala R., Lawande M., Shah G.V. (2012). Central nervous system tuberculosis: Pathophysiology and imaging findings. Neuroimaging Clin. N. Am..

[24] del Saz S.V., Sued O., Falcó V., Agüero F., Crespo M., Pumarola T., Curran A., Gatell J., Pahissa A., Miró J. (2008). Acute meningoencephalitis due to human immunodeficiency virus type 1 infection in 13 patients: Clinical description and follow-up. J. Neurovirol..

[25] Absinta M., Cortese I.C., Vuolo L., Nair G., De Alwis M.P., Ohayon J., Meani A., Martinelli V., Scotti R., Falini A. (2017). Leptomeningeal gadolinium enhancement across the spectrum of chronic neuroinflammatory diseases. Neurology.

[26] Bathla G., Freeman C.W., Moritani T., Song J.W., Srivastava S., Soni N., Derdeyn C., Mohan S. (2020). Retrospective, dual-centre review of imaging findings in neurosarcoidosis at presentation: Prevalence and imaging sub-types. Clin. Radiol..

